# Uncovering the Lipid Web: Discovering the Multifaceted Roles of Lipids in Human Diseases and Therapeutic Opportunities

**DOI:** 10.3390/ijms241713223

**Published:** 2023-08-25

**Authors:** Manoj Kumar Pandey

**Affiliations:** 1 Division of Human Genetics, Cincinnati Children’s Hospital Medical Center (CCHMC), 3333 Burnet Avenue, MLC-7016, Suit R1.019A, Cincinnati, OH 45229, USA; manoj.pandey@cchmc.org or pandeymj@ucmail.uc.edu; Tel.: +1-513-803-1694; Fax: +1-513-636-1321; 2 Department of Pediatrics, University of Cincinnati College of Medicine, 3333 Burnet Avenue, Cincinnati, OH 45229, USA

## 1. Introduction

Lipids, characterized by their hydrophobic nature, encompass a wide range of molecules with distinct properties and functions [1,2]. Fatty acids, the building blocks of lipids, can be saturated or unsaturated, influencing their physical state and biological effects [3,4]. Some examples of fatty acids include palmitic acid, stearic acid, oleic acid, and linoleic acid [5,6,7]. Glycerol, a three-carbon alcohol, serves as the backbone for most lipids and contributes to the formation of triglycerides, the primary form of fat storage through ester bonds [8,9,10]. Sterols, such as cholesterol, play vital roles in cellular processes, including membrane formation, hormone synthesis, and the absorption of vitamins (e.g., A, D, E, and K), which are crucial for various physiological processes, including vision, bone health, immune function, and blood clotting [11,12,13]. Additionally, phospholipids, with their hydrophilic head and hydrophobic tail, contribute to the structure and integrity of cell membranes [14,15,16].

Lipids are further classified based on their function. Structural lipids, such as phospholipids and cholesterol, ensure the integrity and fluidity of cell membranes, thereby facilitating proper cellular function [17,18]. Storage lipids, primarily triglycerides and fatty acids stored in specialized cells called adipocytes and can be mobilized when the body requires energy [19,20,21]. Signaling lipids, including eicosanoids act as chemical messengers and help regulate various cellular activities such as inflammation, blood clotting, and smooth muscle contraction [22,23]. Steroid hormones such as estrogen, testosterone, and cortisol synthesized from cholesterol act as chemical messengers and are involved in a wide range of functions, including growth and development, reproduction, stress response, and metabolism [24,25,26,27,28,29,30].

Dysregulation of lipid metabolism contribute to the formation of neuronal inclusions and the loss of specific neurons in neurodegenerative diseases, including Alzheimer’s [31,32], Parkinson’s [33], and Huntington’s diseases [34,35]. Furthermore, disruptions in lipid homeostasis are associated with chronic inflammation and insulin resistance, contributing to the development of cardiovascular disease and diabetes [36,37].

Overall, lipids are fundamental components of human biology and play diverse and critical roles in maintaining health and cellular function. Understanding the intricate interplay between lipids and disease provides valuable insights into the pathogenesis of various human disorders. The articles selected in this editorial highlight the complex relationship between lipids and disease, emphasizing the importance of further research to uncover novel therapeutic targets and strategies for prevention and treatment. By unraveling the intricate roles of lipids in human diseases, we pave the way for advancements in personalized medicine and interventions that target lipid metabolism to improve patient outcomes.

**A. Unleashing lipid power: Exploring cholesterol and specialized proresolving lipid mediators for cardiovascular Health.**

Cardiovascular disease remains a significant global health concern, with lipids playing a central role in its development and progression [38,39]. In the search for effective preventive measures, studies have turned their attention to understanding the intricate workings of lipoproteins, particularly high-density lipoprotein (HDL) and low-density lipoprotein (LDL) cholesterols. HDL has garnered considerable attention for its ability to perform reverse cholesterol transport, a process crucial for removing excess cholesterol from peripheral vessels and transporting it back to the liver for disposal [40]. Elevated levels of LDL cholesterol, often referred to as bad cholesterol, are well-established risk factors for the development of cardiovascular disease [38,39]. The accumulation of LDL cholesterol in arterial walls leads to the formation of atherosclerotic plaques, contributing to the obstruction of blood flow and the occurrence of heart attacks and strokes [41,42,43]. Additionally, other lipid-related factors such as triglycerides and lipoprotein lipids further contribute to arterial hardening and the onset of arteriosclerosis, stroke, heart attack, and pancreatitis [44,45,46,47,48,49].

The concept of reverse cholesterol transport revolves around HDL’s capacity to shuttle excess cholesterol from peripheral tissues, including artery walls, back to the liver. By facilitating this vital process, HDL helps prevent cholesterol accumulation and the subsequent formation of plaque, which is a major contributor to the development of atherosclerosis. As such, HDL emerges as a vital player in maintaining cardiovascular health [40]. While reverse cholesterol transport is HDL’s primary function, recent research has uncovered additional beneficial properties of HDL cholesterol. HDL has been found to exhibit antioxidative, anti-inflammatory, endothelial, vasodilatory, antithrombotic, and cytoprotective functions [50,51,52,53]. These diverse attributes enhance HDL’s protective effect against cardiovascular disease, extending its impact beyond cholesterol metabolism alone. The multifunctional nature of HDL cholesterol positions it as a potential therapeutic target for interventions aimed at reducing cardiovascular risk.

In recent years, the role of specialized proresolving lipid mediators (SPMs) derived from ω-3 and ω-6 fatty acids, has gained attention as a promising candidate for the management of atherosclerosis. These specialized lipid mediators have exhibited the ability to promote the resolution of inflammation, presenting a unique therapeutic avenue that complements existing interventions. By modulating the immune response and resolving chronic inflammation, SPMs hold the potential to attenuate the progression of atherosclerotic plaques and reduce the associated cardiovascular risks [54,55]. The editorial on this topic aims to focus on the potential of SPMs derived from fatty acids in the treatment and prevention of atherosclerosis and the resolution of inflammation.

**Prioritizing specialized proresolving lipid mediators research and development.** Salazar et al.’s review emphasized the importance of continued investigation and development in the field of SPMs. While the existing evidence regarding their therapeutic potential is promising, several aspects warrant further exploration. Optimal dosing and duration of treatment need to be elucidated, ensuring that the administration of SPMs aligns with patient needs and specific disease conditions. Additionally, understanding potential interactions between SPMs and other medications is essential to maximize their efficacy and safety within comprehensive treatment plans [56].

**Translating promising research into clinical practice.** For the clinical application of SPMs to become a reality, the development of stable and bioavailable formulations is crucial. Overcoming challenges related to the formulation and delivery of SPMs will enable their effective utilization in clinical settings. This requires concerted efforts from researchers, clinicians, and pharmaceutical companies to ensure that SPM-based therapies are accessible and feasible for patients with cardiovascular disease [56].

Lipids are intimately involved in the development and progression of cardiovascular disease, with LDL cholesterol and other lipid-related factors contributing significantly to the pathogenesis of atherosclerosis. However, emerging research on SPMs offers new hope in the fight against cardiovascular disease. By promoting the resolution of inflammation, SPMs provide a novel therapeutic approach that complements existing interventions. The review by Salazar et al. underscores the need for further investigation and development in the field of SPMs, including optimal dosing, duration of treatment, and formulation development [56]. Translating this promising research into clinical practice will enhance our ability to prevent and treat cardiovascular disease, improving patient outcomes and global cardiovascular health.

**B. Unraveling the complexities of lipids: Insights into obesity-related diseases.**

Obesity is a global health concern characterized by an excess of adipose tissue, predominantly composed of triglycerides. Such adipose tissue accumulation of triglyceride not only contributes to chronic inflammation but also leads to insulin resistance, paving the way for the development of fatty liver diseases, both alcoholic and non-alcoholic in nature, termed non-alcoholic steatohepatitis (NASH) and alcoholic steatohepatitis (ASH) [57,58]. The association between obesity, insulin resistance, and altered lipid metabolism extends to type 2 diabetes, the leading cause of death among adults [59,60,61]. Understanding the complex relationship between lipids and these diseases is crucial for effective management and prevention. The editorial on this topic highlights recent studies focusing on the distinct mechanisms and pathways associated with obesity-related diseases, offering valuable insights into potential therapeutic interventions.

**Differential responses in non-alcoholic and alcoholic steatohepatitis.** Dorochow et al.’s study investigated the lipid omics, metabolomics, and immunological responses in mouse models of NASH and ASH. The findings revealed that both conditions resulted in comparable disease severities, including mortality rates, neurological behavior, fibrosis markers, and albumin levels [62]. These similarities in disease progression suggest common mechanisms underlying severe liver damage. However, further investigation is needed to fully elucidate the specific mechanisms responsible for these observed parallels. This study paves the way for a deeper understanding of NASH and ASH and may open new avenues for therapeutic intervention.

**Serum ceramides in chronic hepatitis C virus infection.** HÖring et al. examined the associations between serum ceramide levels and disease markers in patients with chronic hepatitis C virus (HCV) infection. The study revealed that patients with liver cirrhosis exhibited lower levels of specific serum ceramide species compared to those without cirrhosis. Furthermore, direct-acting antiviral therapy demonstrated a more favorable ceramide profile in non-cirrhosis patients but not in cirrhosis patients [63]. These findings highlight the association between serum ceramides, liver cirrhosis, and viral genotype. Understanding the role of ceramides in HCV infection and liver disease progression could aid in the development of targeted therapies for this population.

**Mediator 25 and adipogenesis.** Saunders et al.’s study focused on the functional relevance of Mediator 25 (Med25), a component of the mediator complex involved in relaying signals for adipogenesis. The depletion of Med25 was found to enhance lipid accumulation during the differentiation of adipocytes. The study demonstrated that this increased lipid accumulation was driven by the upregulation of adipogenic master regulators peroxisome proliferator-activated receptor gamma2 (PPARγ2) and CCAAT/enhancer binding protein alpha (C/EBPα) [64]. These findings uncover the role of Med25 in regulating myocardial lipid accumulation and its potential as a therapeutic target for metabolic disorders associated with excessive adipogenesis.

**Sex differences and adipose tissue in novel coronavirus disease.** Thangavel et al. explored the relationship between severe acute respiratory syndrome coronavirus-2 (SARS-CoV-2) viral loads in adipose tissue, immune signaling, and sex differences. The study revealed distinct differences in viral loads between males and females in adipose tissue and lungs. Moreover, the relationship between viral loads in these tissues differed between the sexes. The study also highlighted the differential impact of SARS-CoV-2 infection on immune signaling, lipolysis, and cell death signaling in adipose tissue between male and female mice [65]. This investigation provides a foundation for understanding the complex interplay between fat tissues, immune signaling, and metabolism in patients with novel coronavirus disease (COVID-19), with potential implications for tailored therapeutic strategies.

The recent studies discussed in this editorial emphasize the rich involvement of lipids in obesity-related diseases [62,63,64,65]. Understanding the distinct mechanisms and pathways associated with these conditions is crucial for developing effective therapeutic interventions. The findings emphasize the need for further research to elucidate the underlying mechanisms responsible for disease progression and to explore the potential of targeting specific lipid-related pathways for therapeutic purposes. With continued investigation, we can harness the power of lipid science to combat the rising burden of obesity-related diseases and improve the health outcomes of affected individuals.

**C. Unveiling the complexities of lipid metabolism in cancer: Implications for therapeutic strategies.**

Lipid droplets, cellular organelles involved in lipid trafficking and consumption, play a vital role in various cellular processes, including energy production, protection against oxidative stress, and membrane biogenesis. By sequestering toxic lipids and engaging in a complex relationship with autophagy, lipid droplets help maintain cellular homeostasis and prevent lipotoxic damage [66,67]. In the context of cancer, lipid metabolism assumes even greater significance, as it influences cancer cell growth, survival, and response to therapy [68,69].

Lipid droplets act as vehicles that coordinate lipid trafficking and consumption within cells. These versatile organelles facilitate energy production, protect against oxidative stress, and contribute to membrane biogenesis during rapid cell growth. By sequestering toxic lipids such as fatty acids, cholesterol, and ceramides, lipid droplets safeguard cells against lipotoxic damage. Additionally, the interplay between lipid droplets and autophagy is crucial for maintaining cellular homeostasis. Understanding the intricate mechanisms underlying lipid droplet function in cancer cells provides opportunities for novel therapeutic interventions. The editorial on this topic explores recent studies that highlight the role of lipid droplets, lipolysis, and lipophagy in cancer and highlights their therapeutic implications.

**Lipolysis and lipophagy in cancer.** Lipolysis, the breakdown of lipids, and lipophagy, the degradation of lipid droplets through autophagy, play essential roles in cancer cell growth and survival. Recent studies have highlighted the significance of these processes in modulating cancer progression. Alizadeh et al. and Maan et al. have explored the involvement of lipolysis and lipophagy in cancer cell survival, highlighting their potential as therapeutic targets [68,69]. The dysregulation of lipid metabolism in cancer cells offers opportunities to exploit these processes for developing innovative cancer therapies.

**Targeting nutrient availability for cancer therapy.** Turchi et al. investigated the possibility of targeting nutrient availability as a complementary option for anti-cancer therapy, focusing on breast cancer models. By modifying the diet to be poor in proteins and sulfur amino acids while rich in oxidizable polyunsaturated fatty acids, the study revealed promising results in inhibiting breast cancer cell growth in vitro and in vivo [70]. Targeting the specific nutrient dependencies of cancer cells holds great potential for the development of personalized and effective therapeutic strategies.

**Leptin-mediated regulation of lipid metabolism in breast cancer.** Luo et al. conducted a study to unravel the mechanisms underlying leptin-mediated regulation of lipid metabolism in breast cancer cells. Leptin, a crucial regulator of metabolism and energy homeostasis, has been implicated in cancer proliferation and metastasis. The study demonstrated that leptin activates fatty acid oxidation and inhibits fatty acid synthesis by suppressing the expression of fatty acid synthase in breast cancer cells [71]. These findings not only enhance our understanding of the complex interplay between leptin and lipid metabolism but also identify potential therapeutic targets for breast cancer treatment.

The sophisticated relationship between lipid metabolism and cancer has become increasingly evident, with lipid droplets, lipolysis, and lipophagy emerging as crucial players in cancer cell growth and survival. The studies discussed in this editorial highlight the multifaceted roles of lipid metabolism in cancer and highlight their therapeutic implications [70,71]. Targeting lipid metabolism pathways, nutrient availability, and key regulatory molecules such as leptin holds promise for developing innovative and effective cancer therapies. Continued research in this field will deepen our understanding of the complex interplay between lipids and cancer, paving the way for personalized treatment strategies and improved patient outcomes.

**D. Deciphering lipid dynamics in neuroinflammatory and neurodegenerative diseases.**

Lipids play a vital role in the brain, serving as key components for neural communication, neurogenesis, synaptic transmission, signal transduction, and membrane compartmentalization [72]. Disruptions in specific lipid classes have been linked to the loss of specific neurons and the formation of neurodegenerative disease markers, such as Lewy bodies and Lewy neurites, observed in diseases like Alzheimer’s, Parkinson’s, and Huntington’s diseases [73,74,75,76,77,78,79]. Lipid rafts, another crucial factor in cellular signaling processes, are also implicated in neurodegenerative disorders, causing abnormal protein distribution, toxic cell signaling, and neuropathological events [80,81,82,83]. This editorial highlight recent studies that unravel the intricate relationship between lipids and neuroinflammation and neurodegenerative diseases, providing insights into potential therapeutic targets.

**Modulating neuroinflammation through lipid metabolism.** Haibo et al. explored the role of acyl-CoA:cholesterol acyltransferase 1/sterol O-acyltransferase 1 (ACAT1/Soat1) in LPS-induced neuroinflammation. By inactivating Acat1/Soat1 in myeloid cells and using a selective ACAT1 inhibitor, the study revealed a significant reduction in LPS-induced activation of pro-inflammatory response genes. The findings demonstrated that ACAT1/Soat1 inhibition altered the intracellular fate of Toll-Like Receptor 4 (TLR4), suppressing its pro-inflammatory signaling cascade [84]. This study provides valuable insights into the regulation of neuroinflammation through lipid metabolism, offering potential avenues for therapeutic intervention.

Acyl-CoA:cholesterol acyltransferase 1/sterol O-acyltransferase 1 inhibition and ER-mitochondria connection in Alzheimer’s disease: Disruptions in cholesterol metabolism have been observed in neurodegenerative disorders, including Alzheimer’s disease (AD). Harned et al. investigated the role of ACAT1/SOAT1, a cholesterol storage enzyme enriched at the mitochondria-associated ER membrane (MAM), in reducing amyloid pathology and cognitive deficits in AD mouse models. The study demonstrated that ACAT1/SOAT1 inhibition strengthened the connection between the ER and mitochondria by increasing the number of ER-mitochondria contact sites and shortening their distance. The findings suggest that manipulating local cholesterol levels at the MAM can alter inter-organellar contact sites and underlie the therapeutic benefits observed with ACAT1/SOAT1 inhibition [85]. This study provides valuable mechanistic insights into the potential therapeutic implications of targeting cholesterol metabolism in AD.

**Distinct lipid profiles in malaria-associated cerebral malaria.** Batarseh et al. compared plasma vesicle lipid profiles between mice infected with different strains of plasmodium, focusing on cerebral malaria. The study revealed distinct differences in the lipid composition of plasma vesicles between the two malaria strains. Notably, specific changes in lysophosphatidylcholines, lysophosphatidylethanolamine, and triglycerides were associated with experimental cerebral malaria [86]. These findings feature the lipid alterations associated with cerebral malaria and provide a foundation for further studies on the role of lipids in the pathophysiology of this fatal complication of Plasmodium infection.

**Lipid involvement in alpha-synuclein aggregation and neuroinflammation in Parkinson’s disease.** Hatton and Pandey reviewed the emerging role of various lipids in alpha-synuclein (α-syn) aggregation and the subsequent cellular and humoral immune reactions that trigger neuroinflammation in Parkinson’s disease (PD). Partial glucocerebrosidase enzyme deficiency and the consequent accumulation of glycosphingolipids and α-syn aggregation have been linked to neurodegeneration and memory and motor defects in PD. The study highlights the involvement of triglycerides, diglycerides, glycerophosphoethanolamines, polyunsaturated fatty acids, sphingolipids, gangliosides, glycerophospholipids, and cholesterols in α-syn aggregation and immune activation in PD [76].

Utilizing a genetic and chemically induced glucocerebrosidase targeted experimental mouse model and human cell model of Gaucher disease, we and others have demonstrated that targeting complement 5a (C5a)-C5a receptor1 (C5aR1) axis led to a reduction in glucosylceramide synthesis, production of proinflammatory cytokines and protection from the tissue damage [87,88,89]. These findings suggest that targeting the C5a-C5aR1 axis could hold therapeutic potential for mitigating the lipid induced neuroinflammatory complications in PD. Understanding the role of such complement activation and lipids provide insights into the pathogenesis of PD and potential therapeutic targets.

Overall, the studies discussed in this editorial highlight the complex relationship between lipids and neuroinflammatory and neurodegenerative diseases [76,84,85,86]. From modulating neuroinflammation through ACAT1/SOAT1 inhibition to elucidating lipid profiles in malaria-associated cerebral malaria and α-synuclein aggregation in PD, these studies provide valuable insights into lipid dynamics and their implications for therapeutic strategies. Continued research in this field will deepen our understanding of the complex interplay between lipids and neurological diseases, paving the way for innovative approaches to intervene in disease progression and improve patient outcomes.

## 2. Conclusions

Lipids, comprising fatty acids, glycerol, and sterols, serve as vital components in cellular structure, energy storage, and signaling. While lipids are essential for maintaining normal bodily functions, disturbances in lipid metabolism can contribute to the pathogenesis of various human diseases, i.e., diabetes [90,91], obesity [92,93], cancer [94,95,96], cardiovascular [97,98,99,100,101], malaria [102,103], epilepsy [104], COVID-19 [105,106,107,108], Alzheimer [109], Parkinson [75,76] and lysosomal storage diseases [58]. Understanding the sophisticated interplay between lipids and human health is crucial for developing effective strategies to combat lipid-related disorders. The selection of research and review articles included in this Special Issue provides valuable insights into the pathogenic processes and the role of lipids in human health and disease.

The studies discussed in this editorial focus on the multifaceted roles of lipids in different disease contexts. From the protective effects of HDL cholesterol in cardiovascular disease to the implications of lipid droplets in cancer growth and survival, neuroinflammation, and neurodegenerative diseases, the importance of lipid metabolism cannot be overstated. These studies have highlighted the obscure relationship between lipids and disease, offering insights into potential therapeutic targets and strategies.

By understanding the specific alterations in lipid metabolism associated with different diseases, studies can develop targeted interventions to restore lipid homeostasis and mitigate disease progression. The potential of modulating lipid metabolism, such as targeting C5a/C5aR1 pathway in PD and lysosomal storage diseases [58,87,88,89], ACAT1/SOAT1 inhibition in Alzheimer’s disease [85], or modifying nutrient availability in cancer cells [70], opens new avenues for therapeutic approaches.

Furthermore, the exploration of lipid profiles and lipid-mediated mechanisms in various diseases, such as malaria-associated cerebral malaria [86] and leptin-mediated regulation of lipid metabolism in breast cancer [71], provides valuable insights into disease pathogenesis and potential diagnostic markers.

As our understanding of lipid metabolism continues to deepen, there is a growing appreciation for the elaborate role lipids play in human health and disease. Future research efforts should focus on unraveling the complex mechanisms underlying lipid dysregulation, exploring lipid-lowering strategies, and identifying novel therapeutic targets to combat lipid-related disorders.
